# Using Stochastic Gradient Boosting to Infer Stopover Habitat Selection and Distribution of Hooded Cranes *Grus monacha* during Spring Migration in Lindian, Northeast China

**DOI:** 10.1371/journal.pone.0089913

**Published:** 2014-02-26

**Authors:** Tianlong Cai, Falk Huettmann, Yumin Guo

**Affiliations:** 1 College of Nature Conservation, Beijing Forestry University, Beijing, China; 2 Ecological Wildlife and Habitat Analysis of the Land and Seascape Lab, Biology and Wildlife Department, Institute of Arctic Biology, University of Alaska Fairbanks, Fairbanks, Alaska, United States of America; 3 College of Nature Conservation, Beijing Forestry University, Beijing, China; Institut Pluridisciplinaire Hubert Curien, France

## Abstract

The Hooded Crane (*Grus monacha*) is a globally vulnerable species, and habitat loss is the primary cause of its decline. To date, little is known regarding the specific habitat needs, and stopover habitat selection in particular, of the Hooded Crane. In this study we used stochastic gradient boosting (TreeNet) to develop three specific habitat selection models for roosting, daytime resting, and feeding site selection. In addition, we used a geographic information system (GIS) combined with TreeNet to develop a species distribution model. We also generated a digital map of the relative occurrence index (ROI) of this species at daytime resting sites in the study area. Our study indicated that the water depth, distance to village, coverage of deciduous leaves, open water area, and density of plants were the major predictors of roosting site selection. For daytime resting site selection, the distance to wetland, distance to farmland, and distance to road were the primary predictors. For feeding site selection, the distance to road, quantity of food, plant coverage, distance to village, plant density, distance to wetland, and distance to river were contributing factors, and the distance to road and quantity of food were the most important predictors. The predictive map showed that there were two consistent multi-year daytime resting sites in our study area. Our field work in 2013 using systematic ground-truthing confirmed that this prediction was accurate. Based on this study, we suggest that Lindian plays an important role for migratory birds and that cultivation practices should be adjusted locally. Furthermore, public education programs to promote the concept of the harmonious coexistence of humans and cranes can help successfully protect this species in the long term and eventually lead to its delisting by the IUCN.

## Introduction

The protection of important stopover habitats is essential for conserving migratory bird species. Many long-distance migrants do not have large sufficient energy deposits to complete their migration without proper periods of foraging [1], [2]. In addition, large birds cannot fly non-stop because flight costs increase rapidly with increasing body mass [3]. To migrate successfully, most birds must stop several times on the flyway [4], [5]. Good stopover sites can provide the required energy and roosting areas for migratory birds [6]. The investigation of stopover sites and determination the habitat preferences of migratory birds are crucial for their conservation and habitat management [7], [8], especially for those species facing conservation threats (which is increasingly common today).

**Figure 1:**
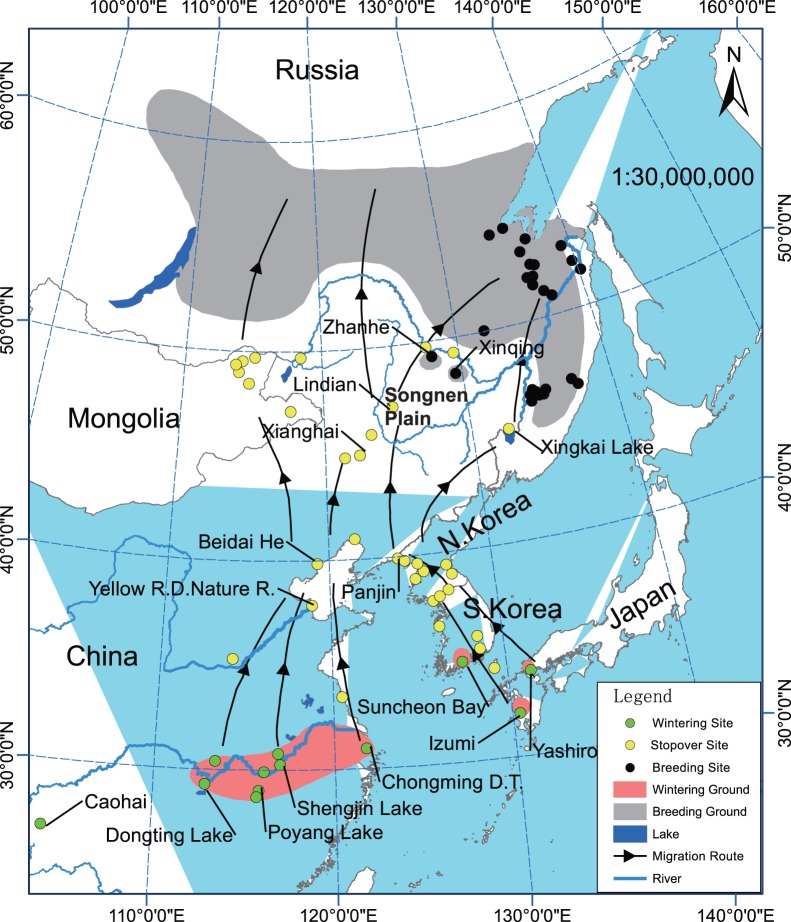
Map of Hooded Cranes distribution and migration routes. Data from *The cranes: status survey and conservation action plan* [9], *Threatened birds of Asia* [10], and field work in recent years.

The Hooded Crane (*Grus monacha*) occurs in northeast Asia and breeds in an area spanning from eastern Siberia to the Chinese Lesser Khingan Range. Its wintering grounds are located in Izumi and Yashiro in Japan, South Korea, and the Yangtze River area in China [9], [10] (Figure 1). Only approximately 11,600 individuals remain throughout the world, and the population is declining [11]. Therefore, the Hooded Crane is recognized as a vulnerable species by the International Union for Conservation of Nature (IUCN) Red List and is listed in Appendix I of the Convention on International Trade in Endangered Species (CITES). The population status of this species does currently not warrant a delisting and requires further attention.

When migrating, most Hooded Cranes stop at several stopover sites on the Korean Peninsula or in China [12]. The western region of the Songnen Plain (Figure 1) receives considerable use by this species due to its abundant food resources. This area of the Songnen Plain is an important network node and stopover site between their breeding and wintering grounds during migration [13]. Lindian, China, is one of the most important stopover sites, and there are records of large numbers of this species in the Songnen Plain. During our 10 years of field work to survey the population of Hooded Cranes, we found over 4,000 individuals or c. 34% of the world population, stopping in this region during each migration. However, this species is faced with threats such as habitat loss and hunting. The conservation of cranes is closely tied to the protection of their habitat, including breeding and wintering grounds as well as stopover sites [14]. Without proper conservation measures, the stopover sites will become weak links in the chain of a successful migration that, if broken, would likely signal the end of the wild crane populations that rely on these sites [15]. Thus, it is necessary to study and protect this area for the effective conservation management of Hooded Cranes, and there are national and international legal mandates to do so.

Although many studies of the habitat selection of other crane species have been conducted [16]–[19], very little is known about Hooded Cranes, and published refers only to wintering populations [20], [21]. There is sparse information regarding stopover sites, and this work has been limited to observations and descriptive studies [22]. In addition, we found that the use of stopover site was not constant over recent years, and even less is known about other stopover sites in this area. The effective conservation of stopover sites requires a thorough understanding of what makes a site important [6]. Yet, despite Lindian’s relatively large numbers and its importance for Hooded Crane migration, this area has not been assessed and quantified.

To gather such information in a robust manner, we used a progressive resource selection function (RSF) approach. An RSF is any mathematical function estimating the selection of resources (in space and time) from binary observations of resource units (either used-vs.-unused or used-vs.-available) [23], [24]. RSF has been widely utilized for habitat assessments [25] and applied to compare sites used by wildlife to unused sites [23]. The models are usually based on generalized linear algorithms [24], and information criteria such as AIC and BIC are commonly used to select the parsimonious model from a set of alternative plausible models [26]. Here, we used a more convenient and powerful algorithm called stochastic gradient boosting (SGB) [27] to analyze the habitat preferences of Hooded Cranes. To understand their preferences for the various habitats, the stopover habitat was subdivided into roosting site, daytime resting site, and feeding site habitats. This methodology offers a more sophisticated approach to studying the ecology of this species and its habitat than most previous studies on cranes, which have tended to focus on one aspect but have not used a multivariate approach that allows for a more nuanced interpretation of the data.

A species distribution model (SDM) was developed in this study to quantify the Hooded Crane daytime resting site distribution in our study area. Such a model is useful for conservation management because it can be used to consistently predict locations where species are likely to occur in areas that have been poorly surveyed or lack such information [28]. This type of research has a long history, and the recent growth of such work has been spurred by developments in geographic information systems (GIS) and related technologies, which have resulted in both a larger amount of available digital data and a wider variety of tools for analyzing these data [29]. Some algorithms, such as maximum entropy, neural networks, and classification and regression trees (CARTs), are commonly applied in the development of SDMs [30]. However, we used the SGB algorithms (TreeNet), which have several advantages: not only are they able to automatically select the important predictor variables (i.e., *a priori* variable selection or data reduction is not required), they and the users can also learn and apply relationships very quickly on most datasets, allowing us to focus on the underlying biological questions [31]. SGBs are based on boosting, and those used here employ bagging [27] to achieve the best-possible predictions with high performance (e.g., the receiver operating characteristic (ROC) and confusion metrics).

The objective of our study was to address several questions regarding habitat selection by Hooded Cranes. i) What types of habitat do cranes prefer? ii) Which environmental factors and conditions in Lindian have made this region such an important stopover site for this species? iii) Is there a daytime resting site in our study area? Answering these questions will help address the ultimate question: how can we conserve this species in this area?

## Methods

### Ethics Statement

This research was conducted in strict accordance with animal care permits issued by China’s State Forestry Administration. The study was conducted on public land with permission from the local government and residents. No further specific permissions were required for our study. The field work did not involve endangered or protected species and did not require permits from an Institutional Animal Care and Use Committee (IACUC) or equivalent animal ethics committee because there was no physical sampling or other potentially detrimental activity toward the animals. In all of our field work, care was taken to minimize any negative impact on the welfare of the animals involved, including the selection of appropriate methods for monitoring the cranes and surveying habitat.

### Study Area

**Figure 2:**
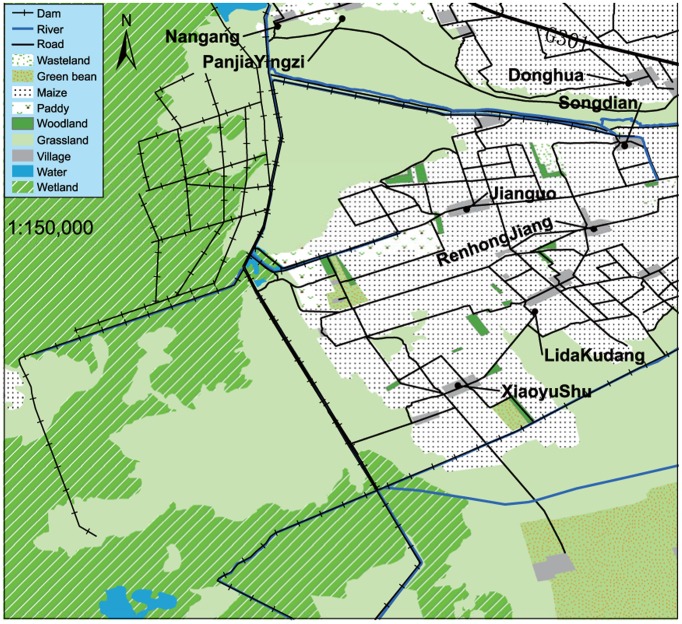
Map of the study area and habitat. This map was created in ArcGIS 10.1 by combining the visual interpretation of a remote sensing image (pixel size resolution of 2×2 m, downloaded from Google Maps; image taken in September, 2010) and our field observations.

We conducted this study in Lindian, northeastern China (Figure 1), in the spring seasons of 2012 and 2013. Lindian (46°44′N∼47°29′N, 124°18′E∼125°21′E) is located in Northeast China at the Wuyu’er River on the west Songnen Plain where there are large areas of wetland, grassland, and farmland that are highly suitable to water birds. This area is one of the major grain producing regions of China. The grain crops cultivated by resident farmers consist of planted maize, rice, and green beans. Each autumn, the mechanical harvest leaves a significant quantity of grain in the fields. This leftover grain is a primary food resource for migratory birds, making this area a favorable stopover site for Hooded Cranes, where they can recover after long-distance travel. Thus, it appears that these populations are subsidized by the farming practice. Our study area comprised 30,000 ha and encompassed the largest known stopover site on the western side of Lindian (Figure 2).

### Data Collection

We divided the area into three types of habitat based on the utilization by migratory Hooded Cranes. The feeding site was situated in farmland and used for foraging by cranes. The daytime resting site was used by crane families to retreat there when they were disturbed at their feeding sites. They used the area for resting, maintaining themselves, and preening, although they foraged less in this area than in the feeding site. The roosting site was used for resting at night. We recognized such sites by finding dropped feathers and feces, and these were confirmed by using infrared cameras (LTL 5210A, China; Mode: Camera+Video; Automatic shoot: 3 pictures; Interval: 30 s; Sense Level: normal).

**Figure 3:**
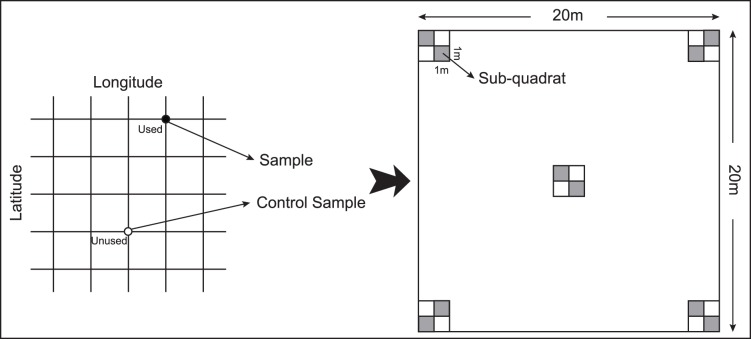
Schematic diagram of the quadrat and sub-quadrat selection.

In the field, we used survey quadrats to investigate the habitat in a quantitative manner. Prior to our field work, a 10×10 (latitude and longitude) gridded map (WGS-1984) was drawn to cover the entire study area. In the field, we found the intersection points (i.e., the center points of the squares) of the grid using a GPS receiver (eXplorist 500, USA). After an intersection point was used by the Hooded Cranes, we regarded this place as a sample point (used). Otherwise, we regarded it as control point (unused). From every sample point and control point, we placed 10 sub-quadrats (1×1 m) within a wider quadrat (20×20 m) (Figure 3). We evaluated each sub-quadrat for the vegetation index value and the abundance of food (Table 1) and used the average of the 10 quadrats as the representative ecological characteristics of that sample point (or control point).

**Table 1:** Predictor variables used for developing habitat selection models in TreeNet.

Variable	Habitat Type	Description
DISROAD	R,D,F	The distance from the sample (or control) to the nearest road (m)
DISRIVER	R,D,F	The distance from the sample (or control) to the nearest river (m)
DISDAM	R,D,F	The distance from the sample (or control) to the nearest dam (m)
DISVILLAGE	R,D,F	The distance from the sample (or control) to the nearest village (m)
DISFARM	R,D	The distance from the sample (or control) to the nearest farmland (m)
DISWETLAND	D,F	The distance from the sample (or control) to the nearest wetland (m)
PLANT	R,D,F	The number of plant species in the sample (or control)
HEIGHT	R,D,F	Average height of plants in the sample (or control) (cm)
COVERAGE	R,D,F	Average coverage of plants in the sample (or control) (%)
DENSITY	R,D,F	Average density of plants in the sample (or control) (m^-2^)
FOODTYPE	R,D,F	All types of food in the sample (or control)
FOODQUA	R,D,F	Average quantity of food in the sample (or control) (seeds/m^2^)
WATERDEEP	R	Average depth of water in the sample (or control) (cm)
OPENWATER	R	Open water area in the sample (or control) (m^2^)
LEAVES	R	Average coverage of deciduous leaves in the sample (or control) (%)

“R”, “D”, and “F” correspond to roosting sites, daytime resting sites, and feeding sites, respectively.

For samples and control points, we used a desktop analysis to record the distances to road, river, dam, village, farmland and wetland (Table 1). Based on the study area map (Figure 2), we calculated the distance variable using the Euclidean distance tool in ArcGIS 10.1. We then extracted the data using the freely available Hawth’s Tools Geospatial Modeling Environment (GME) (http://www.spatialecology.com/htools) [32]. A total of 95 sample points and 90 control points were surveyed in the field during 2012 and 2013.

### Data Processing

Our models are based on the SGB algorithm (TreeNet) designed by Jerome Friedman [27] and found in Salford Predictive Modeler (SPM 7.0) (http://www.salford-systems.com), which is available to researchers in a trial version (here we used the EWHALE lab license).

We employed used-vs.-unused data, a type of presence/absence data using the sample points and control points, to generate RSF models. This allowed us to assess the differences in habitat preferences as a snapshot in space and time. We assigned a value of zero to sites where the Hooded Crane was absent and a value of one to sites where it was present. A wider set of predictor variables (Table 1) was selected *a priori* using known and hypothesized habitat relationships based on the ecology of the species [33]. Data were then imported into SPM 7.0 from an Excel table and analyzed for each of the three types of habitat using identical settings. To obtain the best possible model fits and prediction accuracies, we used the following settings: classification tree, tree size of 1,000, balanced sample weights, and best model chosen by ROC [34]. This resulted in three habitat selection models: i) the roosting site selection model, ii) the daytime resting site selection model, and iii) the feeding site selection model.

After the RSFs were performed, a SDM was developed based on the presence-available modeling approach used to predict daytime resting site occurrences in accordance with design II in Manly et al. [24]; [35]. To develop the model, we used five predictors overall: distance to wetland, distance to farmland, distance to road, distance to river, and distance to village. Those layers were established using the Euclidean distance tool (ArcGIS 10.1) with the 50 m distance based on the map (Figure 2) extracted from the remote sensing image. We also extracted data from environmental layers at the sample points and control points using Hawth’s Tools. The model was then constructed using a classification and the balanced class weights option to account for the unequal sample sizes of presence and available points commonly used in RSFs. We obtained the predictive map using an approach employed with the Gyrfalcon (*Falco rusticolus)* nest distributions in Alaska [36]. In our study, we created a high-resolution point lattice grid of 161,157 regularly spaced points; the cell size was the same as that used for sampling, i.e., approximately 20×20 m. We extracted the relative occurrence of 40 test points (the presence points were collected in 2013) and 31 control points (the absence points were collected in 2012) from the relative occurrence index (ROI) map in Hawth’s Tools. According to the ROI of the test points and control points, we created a boxplot in R 2.13.0 to assess the accuracy of SDMs.

In addition, we used Statistica 6.0 to make specific inferences based on statistical testing. Data were compared using a one-way analysis of variance (ANOVA). Spearman rank correlations were used to test associations of data. Significance was accepted at P≤0.05.

## Results

### Roosting Site Selection

**Figure 4:**
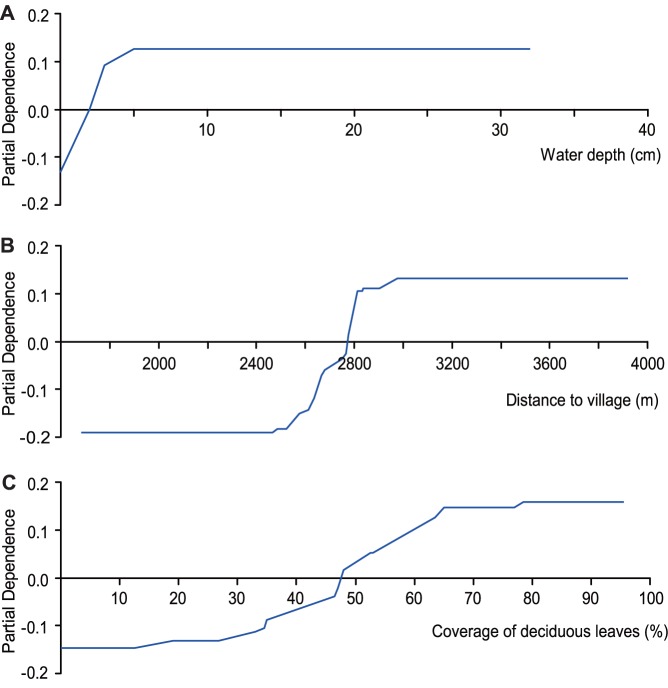
Partial dependence plots for the predictor variables employed in the roosting site selection model; (A) water depth (cm), (B) distance to village (m), and (C) coverage of deciduous leaves (%).

After the data were technically cleaned and formatted, we ran small sample sizes in TreeNet to generate a total of 249 optimal trees from 1000 possible trees. The ROC curve indicated the model discrepancy (accuracy). We then integrated the area under the curve (AUC) to assess the model performance or predictive power [37]. From the result of model, we found that the roosting site selection model achieved a high AUC value (learning/training = 0.98/0.80), indicating a relatively high discrimination and prediction accuracy. For each predictor variable, TreeNet also provided a relative importance score (Table 2), which describes the individual contribution of each predictor in explaining the response variable in the tree models [34]. The model showed that the five most important predictors were the water depth, distance to village, coverage of deciduous leaves, open water area, and density of plants. However, two variables (food quantity and type of food) did not contribute to the model. In addition, we obtained the response curves of these predictors. The response curve shows the relative index as a function of the predictor in the context of the multivariate model: a positive partial dependence indicates preference, and a negative partial dependence indicates avoidance. Detailed data on the predictors indicate that the Hooded Cranes selected the roosting site where water was between 2 to 32 cm deep (Figure 4A), where the distance to the village was greater than 2,760 m (Figure 4B), and where the coverage of deciduous leaves was greater than 48% (Figure 4C).

**Table 2:** The importance ranks of the variables used to predict Hooded Crane habitat selection in TreeNet (the roosting site, the daytime resting site, and the feeding site).

Roostingsite	Score	DaytimeResting Site	Score	Feedingsite	Score
WATERDEEP	100.0	DISWETLAND	100.0	DISROAD	100.0
DISVILLAGE	87.1	DISFARM	45.0	FOODQUA	97.4
LEAVES	61.0	DISROAD	33.4	COVERAGE	87.8
OPENWATER	57.7	DISRIVER	29.6	DISVILLAGE	84.8
DENSITY	53.2	HEIGHT	28.6	HEIGHT	75.7
HEIGHT	45.7	COVERAGE	26.7	DENSITY	75.4
DISROAD	45.4	DENSITY	25.5	DISWETLAND	65.8
COVERAGE	41.1	DISVILLAGE	24.9	DISRIVER	60.0
DISRIVER	34.1	PLANT	21.1	PLANT	33.0
DISDAM	26.4	FOODQUA	21.1	FOODTYPE	20.1
PLANT	22.8	FOODTYPE	3.8	DISDAM	0
DISFARM	21.2	DISDAM	0		
FOODQUA	0				
FOODTYPE	0				

### Daytime Resting Site Selection

**Figure 5:**
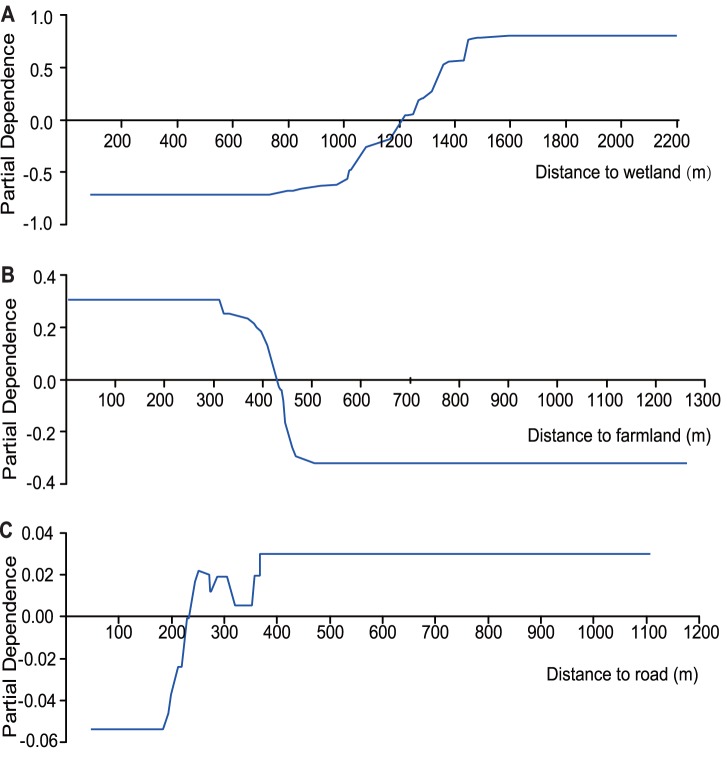
Partial dependence plots for predictor variables employed in the daytime resting site selection model; (A) distance to wetland (m), (B) distance to farmland (m), and (C) distance to road (m).

When the best daytime resting site selection model was constructed, TreeNet created 247 optimal trees and once again obtained a high AUC value (learning/testing = 0.99/0.93). Although this is an internal running metric and was not field tested, it nevertheless indicates that the model was highly accurate and had a good performance. A total of 12 variables were utilized to create the model, although one variable did not contribute (distance to dam). The distance to the nearest wetland received by far the highest rank, and distance to farmland ranked second followed by distance to road (Table 2). The preferred habitat can be described as being more than 1,200 m from a wetland (Figure 5A), less than 445 m from farmland (Figure 5B), and more than 225 m from a road (Figure 5C). Habitats with these characteristics would be preferred by Hooded Cranes for daytime resting sites.

### Feeding Site Selection

**Figure 6:**
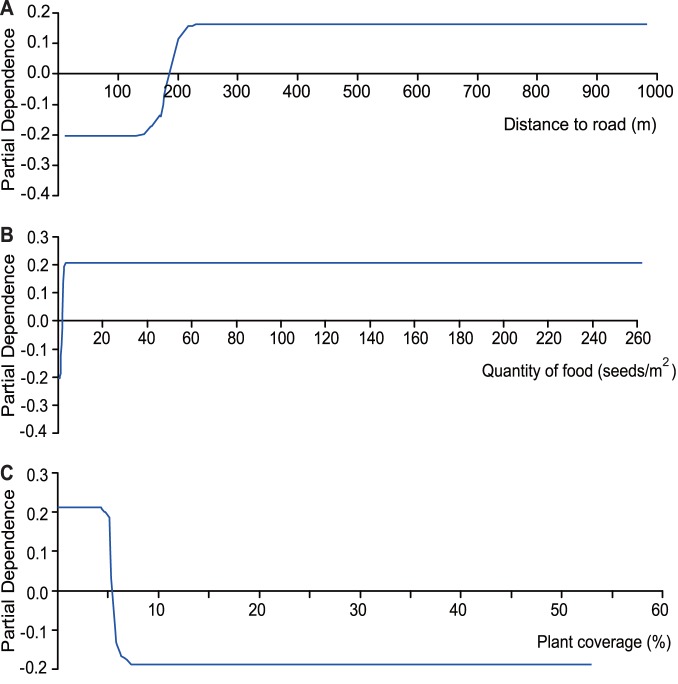
Partial dependence plots for the predictor variables employed in the feeding site selection model; (A) distance to road (m), (B) quantity of food (seeds/m^2^), and (C) plant coverage (%).

We used 11 variables to develop the feeding site selection model, and 800 optimal trees were created in TreeNet. This model also had a good initial performance with a high AUC value (learning/testing = 1/0.81). The importance ranking shows that 8 of the 11 variables make greater contributions to the model, with one variable not contributing (distance to dam). The distance to road and food quantity contributed highly to the model, as did plant coverage and distance to village, followed by plant height, plant density, distance to wetland, and distance to river. The most important variables were distance to road and quantity of food, as they both scored above 90. The partial contribution of the variable values to the model is shown in the single variable plots in Figure 6. Hooded Cranes occurred more often where the distance to a road was greater than 185 m (Figure 6A). The Hooded Crane was also more likely to occur in areas with a food abundance of more than 1.5 seeds/m^2^ (Figure 6B). Plant coverage of less 5.5% had a positive influence on the presence of Hooded Cranes (Figure 6C), whereas a coverage greater than 5.5% had a negative influence.

### Predicting Daytime Resting Site Distribution

**Figure 7:**
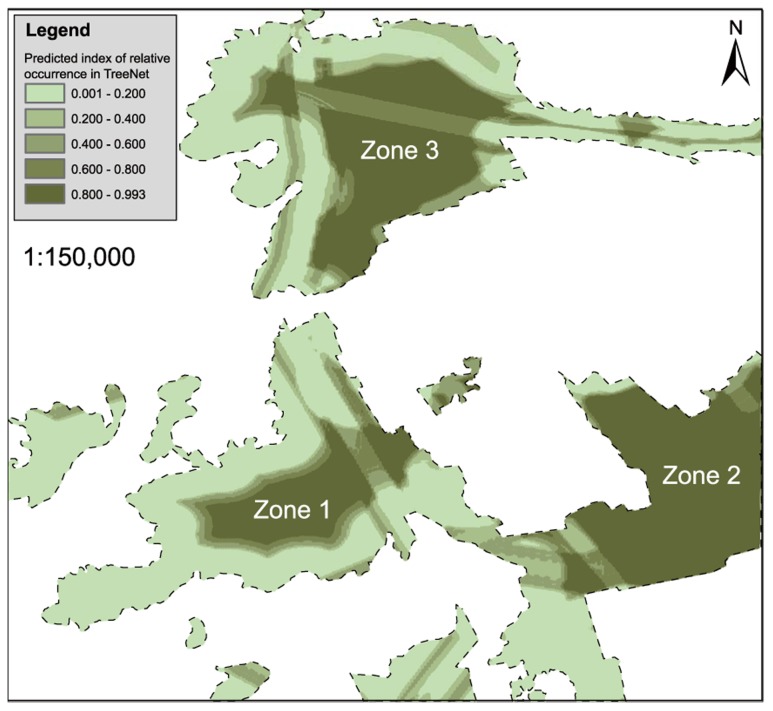
A prediction model map of Hooded Crane daytime resting sites in the study area.

The optimized TreeNet model contained 310 statistical trees and predicted the Hooded Crane occurrence at a daytime resting site (Figure 7). This model also obtained a very high AUC value (learning/testing = 0.99/0.91), indicating a high prediction accuracy for the data and as well as the software. Based on the prediction map, we found that the potential daytime resting sites are distributed within the grasslands. Approximately 45% of the study area was predicted to have an ROI <20%, and 39% of the study area was predicted to have an ROI >60% (Table 3). Cranes were predicted to occur most often (>80%) in the southwest (Zone 1), southeast (Zone 2), and northern (Zone 3) regions of the study area. The map appears somewhat banded because of the road effects, although this is to be interpreted in the context of biological habitat and can be smoothed out in subsequent model runs. Here we provide a robust proof of concept and suggest that the method may be applied to larger areas and other migration hotspots.

**Table 3:** Area of each prediction level and its percentage representation in the study area based on the relative occurrence index (ROI).

Predicted ROI (%)	Area (ha)	Accounts for the percentage of the study area (%)
0∼20	4,709.4	45
21∼40	863.9	8
41∼60	870.8	8
61∼80	739.9	7
81∼100	3,417.2	32

## Discussion

In this study, we used a TreeNet-based implementation of stochastic gradient boosting to develop three habitat selection models and an SDM. These represent the habitat preferences of Hooded Cranes using advanced model analysis methods for the first time and display the distribution of Hooded Cranes at stopover sites in Lindian, China. This study area is of great importance because it represents the heart of the grain production in China and at the same time hosts species of international relevance according to the IUCN. In addition, our study evaluated a new model in the study of habitat selection. This new statistical machine-learning approach can quantify habitat preference, which is performed as an RSF, and quantitatively describe the ecological niche for this species of national and international conservation concern. Because we focused on habitat preference and distribution, in the following sections, we also discuss how the environmental variables influenced the obtained results, propose underlying mechanisms, explore the importance of Lindian for migratory birds, and consider how to conserve this species in a balanced way. In addition, we evaluate the advantages of our models.

### Roosting Site Selection

A suitable roosting site not only retains a desirable temperature for birds but also protects them from predation [38] and other threats. This is because the period of fasting often coincides with peak thermostatic demands, which suggests that there may be strong selective pressures to minimize thermoregulatory stress by selecting appropriate nocturnal roosting sites [39]. In addition, cranes have difficulty perceiving potential dangers during the night because of their poor vision [40]. Therefore, safety and temperature are the key features for ideal roosting sites [41], [42]. Our models clearly show this relationship and quantify these features and proxies for other characteristics. Overall, a shallow-water marsh wetland appears to be of key importance for the presence of the cranes. This species appears to prefer marsh wetlands that have shallow but open water, are far from villages, and have higher plant density and deciduous cover.

### Daytime Resting Site Selection

Daytime resting sites have been widely studied in mammals such as raccoons (*Procyon lotor*) and wild boar (*Sus scrofa*) [43], [44] but are not yet well understood for birds. Some studies have examined feeding sites but did not explicitly define and recognize daytime resting sites or study their function. As a transitional area between feeding and roosting sites, we found that the daytime resting sites played an important role in stopovers and that cranes spent most of their time during the day in these areas. Thus, we defined the daytime resting site as a specific type of habitat for this study, and we found it to be an appropriate classification of crane habitat, meriting further study.

Our model revealed that this habitat was typically located further away from wetlands and roads but closer to farmland (Figure 5) and generally consisted of harvested grassland locations between wetlands and farmland. Interestingly, we revealed that the cranes also forage on grasslands, even though the food in these areas was not as rich as in the farmland (*F_1, 62_* = 4.99, *P*<0.01, one-way ANOVA). In addition, we found that every patch of farmland used by cranes had some grain left that had not been eaten, indicating that the harvested farmlands have sufficient food for this species. Thus, the question arises: why do cranes spend so much time foraging in grassland instead of farmland? Based on our work, we hypothesized that i) the cranes regarded the daytime resting site as a supplementary feeding site when the farmland was disturbed, and ii) the daytime resting site has specific food the cranes need, such as readily digestible food or higher-energy food for juveniles. Invertebrates, i.e., spiders and beetles, may be one such food item found in our study area that occurs more frequently in grassland than in farmland. Such questions should also be addressed in future studies. The cranes foraging in the daytime resting sites were mainly families of adults and juveniles, in support of our second hypothesis.

### Feeding Site Selection

Food is one of three basic requirements for wildlife (together with water and shelter) [45] and provides the nutrition and energy that allows wildlife to survive and reproduce. In the course of foraging, birds routinely choose among habitats or feeding sites that differ in both their net energetic rate of return and in the risk of death to the forager [46]. Arguably, birds prefer to select habitats that provide both a high-energy return and safe shelter as feeding sites. Feeding-danger trade-offs are central to decisions made by such foragers [6].

In this study, farmland appeared to be crucial for Hooded Crane foraging. Food abundance may be an external factor influencing the stopover strategies of migratory birds [47]. Food abundance determines the foraging efficiency, as more food allows more energy to be obtained per unit time. Compared with daytime resting and roosting sites, feeding sites had the most abundant food (*F_2,92_* = 17.08, *P*<0.001). This can allow Hooded Cranes to take in more energy in a relatively short period of time and reduce the foraging time in the fields, thereby reducing the risk of predation. We also calculated the availability of the feeding sites and the habitat selection index [16] for Hooded Cranes in each type of feeding sites (Table 4). It was calculated as the percentage of flocks using a given type of feeding area divided by the percentage of the available area of the same type. Although rice paddies had the highest selection index, maize stubble was the most important feeding site because maize stubble had the largest area (80%), and a large proportion of Hooded Cranes (76.7%) chose to forage on maize stubble. The habitat selection index was significantly correlated with food abundance (*r* = 0.79, *P*<0.01) but did not depend on the availability of the feeding area (*P* = 0.20). Thus, Hooded Cranes did not use the available habitat types in proportion to their availability but showed a distinct preference. This finding is similar to those for other species such as Red-crowned Cranes *G. japonensis* [18] or for instance Brown Kiwi *Apteryx australis* [48]. The phenomenon of habitat selection index not in proportion to their availability may reveal that species cannot make full use of all resources in nature, but that human management of wildlife and habitat may benefit a given species and even lead to population increases.

**Table 4:** The habitat selection index for Hooded Cranes in each type of feeding sites.

Type of feeding area	Average availability (%)	Number of flocks	Percentage of flocks (%)	Food (seeds/m^2^)	Selection index
Maize stubble	80.0	46	76.7	4.06	0.9588
Rice paddy	13.2	11	18.3	8.16	1.3864
Green bean field	5.8	3	5.0	6.06	0.8621
Uncultivated	1.0	0	0	0	0

The habitat selection index was calculated as the percentage of flocks using a given type of feeding area divided by the percentage of the available area of the same type.

### Daytime Resting Site Distribution

The use of ROC curves to assess the accuracy of models has been frequently applied in studies of species distribution, *i.e*., Gyrfalcon [36] and White Stork *Ciconia ciconia* [31]. The SDM that was developed in our study had high prediction discrimination (accuracy) for the model, as demonstrated by its high AUC values. The best way to assess model accuracy and its generalizability in space and time is to determine whether it can make accurate predictions elsewhere, with alternative data, and into the future [49]. In this study, we used the second method to assess whether our model actually has a high accuracy. We developed the SDM based on the data collected in 2012. Our field work showed that daytime resting sites were located in the southwest (Zone 1) region of the study area but not in the southeast (Zone 2) or north (Zone 3). In contrast, Hooded Cranes were not present in the southwest (Zone 1) in the spring of 2013 but mainly stopped over in the southeast (Zone 2), with a few cranes using the northern section (Zone 3).

The use of field observations to assess the accuracy of models may be suitable for small areas, but for large areas, sampling a series of points to test presence and absence may be difficult. Therefore, we sampled 40 presence points to test the model, and the actual results confirmed the model. The boxplot based on the relative occurrence of the 31 control points and 40 test points extracted from the ROI map reveals that test and control points indeed have significant differences (*F_1,69_* = 178.52, *P*<0.001, one-way ANOVA). This analysis provides an alternative measure to confirm that our model has a high accuracy.

### Model Advantages

Our models, based on the SGB algorithm to reveal habitat preference and species distribution, have several advantages. First, our non-linear model is quantitative, fast, and convenient and provides insightful habitat response curves. Compared to traditional linear modeling, the machine learning models such as SGB produce highly accurate predictions, perform faster, and are more informative [50], [51]. Furthermore, non-linear modeling can handle complex datasets without the requirements and assumptions of linear models [50]. Second, our study shows that SGB performs well on the small scale: all models had highly accurate AUC values (>0.8) and were confirmed by field observations. Generally, the SGB provided better and more stable predictions than other algorithms on a broad scale [52]. Finally, our study can be used as an example of a rapid assessment to identify the best predictive model for avian habitat selection. This work not only contributes to an empirical, modern, and quantified understanding of Hooded Crane habitat preferences in stopover sites, but it also provides a distribution map that can help in the development of management strategies for this threatened species. In addition, we provide a theoretical basis for the formulation of a truly science-based conservation program, which could serve as a general template in China and for the flyway.

### Conservation Considerations

A conceptual framework was proposed by Mehlman *et al.* [53] to prioritize stopover sites according to their capacity to provide the necessary resources that migratory birds require for successful migration. The authors suggest that the criteria to classify and prioritize stopover sites for conservation should include three aspects: i) ecological context (including the proximity to ecological barriers and degree of spatial isolation); ii) intrinsic characteristics (e.g., food abundance and protection from predators); and iii) migrant use (e.g., relative abundance, including frequency and consistency of use as a stopover site). Based on this framework and on the inherent advantages of Lindian, we suggest that our study area is very important to migratory birds.

First, the location is very advantageous. Lindian is situated in the East Asian flyway (Figure 1) west of the Zhalong Nature Reserve and east of the Songnen Plain. This area has high habitat heterogeneity because it is located in a transitional zone of farmland, grassland, and wetland where there is greater species diversity than could be found in a single-habitat ecosystem [54]. This area is currently the major grain production region in China, and 126,667 ha of maize and 11,667 ha of rice were planted in 2012 (http://www.dqdaily.com/ztxw/quxpd/2013-01/28/content_1400536.htm). A substantial amount of grain remains after mechanical harvesting and then becomes a primary food for migratory birds in autumn and spring. Moreover, the marsh wetland also supplies mollusks and fishes for certain cranes and storks. Finally, this region is also well-utilized as a stopover site by certain migratory waterfowls. Satellite tracking revealed that this area has received considerable use by cranes, storks, and ducks [14], [55]–[57], which stopover at Lindian for several days or more, and some of these birds are even breeding in this area.

In this study, we showed that wetland, grassland, and farmland were preferred by Hooded Cranes as stopover sites and were essential to crane foraging, resting, and roosting. However, the conflict between cranes and local farmers has become increasingly acute with farmland expansion and habitat loss. Hence, conservation measures to protect the species and its habitats should include the following. i) Local education programs are urgently required to promote awareness and public stewardship of the cranes. The concept of harmonious coexistence of humans and cranes reflecting a harmonious coexistence between nature and mankind [58], already a very popular sentiment in Japan, should be developed locally. In Hokkaido, cranes co-exist relatively well with humans. Humans provide food to the Red-Crowned Crane, which has increased the population of this species in recent years [9]. ii) Farming methods should be appropriately adjusted to satisfy crane migration. Machine harvesting should be encouraged and widely used because it contributes to crane foraging. The harvester not only leaves a lot of grain behind but it can also reduce the vegetation height and cover. Furthermore, straw burning should be strictly banned after the harvest because it destroys the scattered grain, removing this food source. iii) The local government should subsidize the losses to local farmers that are caused by foraging cranes. Luo et al. [59] found that farmers with low income are reluctant to bear the losses caused by cranes. Thus the local residents may drive the Hooded Crane from their arable land and disturb them if they see the cranes feeding on their crops. Subsidization can ease the conflict between humans and cranes and contribute to the protection of the Hooded Cranes.
