# Correction: Using Stochastic Gradient Boosting to Infer Stopover Habitat Selection and Distribution of Hooded Cranes *Grus monacha* during Spring Migration in Lindian, Northeast China

**DOI:** 10.1371/journal.pone.0097372

**Published:** 2014-05-05

**Authors:** The PLOS ONE Staff

The publisher apologizes for the following errors:

An error occurred in Figure 1 during the conversion process. Please see the corrected figure here.

**Figure 1:**
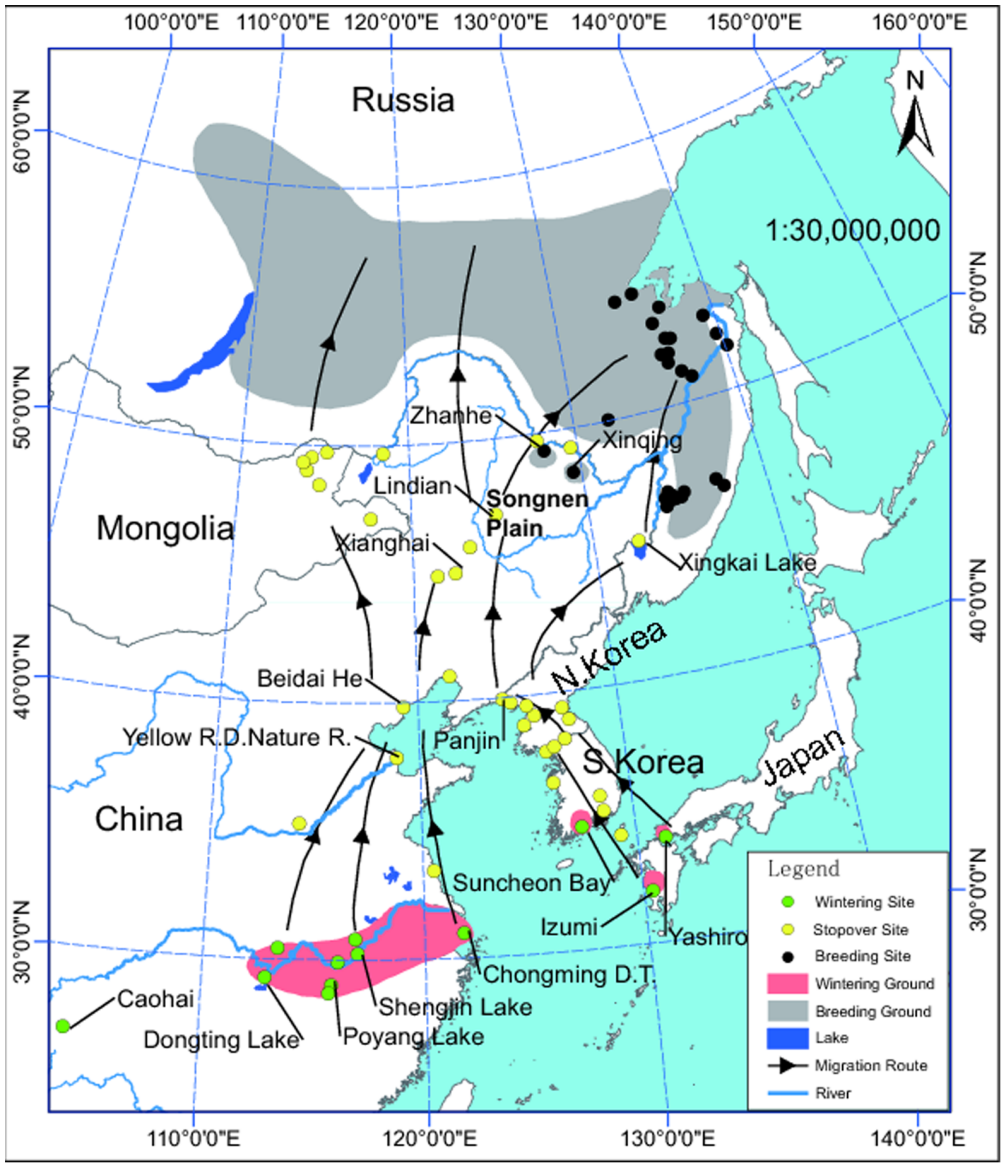
Map of Hooded Cranes distribution and migration routes. Data from *The cranes: status survey and conservation action plan* [9], *Threatened birds of Asia* [10], and field work in recent years.

Units are missing in the second sentence of the second paragraph of the Data Collection section. The correct sentence is: “Prior to our field work, a 10"x10" (latitude and longitude) gridded map (WGS-1984) was drawn to cover the entire study area.”
